# A Cognitive Behavioral Therapy Chatbot (Otis) for Health Anxiety Management: Mixed Methods Pilot Study

**DOI:** 10.2196/37877

**Published:** 2022-10-20

**Authors:** Yenushka Goonesekera, Liesje Donkin

**Affiliations:** 1 Department of Psychological Medicine The University of Auckland Auckland New Zealand; 2 Department of Psychology and Neuroscience Auckland University of Technology Auckland New Zealand

**Keywords:** health anxiety, conversational agent, illness anxiety disorder, COVID-19, iCBT, user experience, anthropomorphism

## Abstract

**Background:**

An increase in health anxiety was observed during the COVID-19 pandemic. However, due to physical distancing restrictions and a strained mental health system, people were unable to access support to manage health anxiety. Chatbots are emerging as an interactive means to deliver psychological interventions in a scalable manner and provide an opportunity for novel therapy delivery to large groups of people including those who might struggle to access traditional therapies.

**Objective:**

The aim of this mixed methods pilot study was to investigate the feasibility, acceptability, engagement, and effectiveness of a cognitive behavioral therapy (CBT)–based chatbot (Otis) as an early health anxiety management intervention for adults in New Zealand during the COVID-19 pandemic.

**Methods:**

Users were asked to complete a 14-day program run by Otis, a primarily decision tree–based chatbot on Facebook Messenger. Health anxiety, general anxiety, intolerance of uncertainty, personal well-being, and quality of life were measured pre-intervention, postintervention, and at a 12-week follow-up. Paired samples *t* tests and 1-way ANOVAs were conducted to investigate the associated changes in the outcomes over time. Semistructured interviews and written responses in the self-report questionnaires and Facebook Messenger were thematically analyzed.

**Results:**

The trial was completed by 29 participants who provided outcome measures at both postintervention and follow-up. Although an average decrease in health anxiety did not reach significance at postintervention (*P*=.55) or follow-up (*P*=.08), qualitative analysis demonstrated that participants perceived benefiting from the intervention. Significant improvement in general anxiety, personal well-being, and quality of life was associated with the use of Otis at postintervention and follow-up. Anthropomorphism, Otis’ appearance, and delivery of content facilitated the use of Otis. Technical difficulties and high performance and effort expectancy were, in contrast, barriers to acceptance and engagement of Otis.

**Conclusions:**

Otis may be a feasible, acceptable, and engaging means of delivering CBT to improve anxiety management, quality of life, and personal well-being but might not significantly reduce health anxiety.

## Introduction

Given the importance of physical health to survival, it is natural and adaptive for people to be vigilant and fearful about their health, engage in health-promoting behaviors, and scan for health threats [1,2]. However, when this fear is associated with functional impairment and distress, it is termed health anxiety [3]. Defined as the persistent worry about illness based on the misinterpretation of bodily symptoms, health anxiety is a criterion of illness anxiety disorder. The tendency of health-anxious individuals to misinterpret typically benign symptoms as a sign of infection [4,5] such as those of COVID-19 (eg, fever, cough, fatigue) likely explains the reported increase in health anxiety during the COVID-19 pandemic [6].

Health anxiety drives the avoidance of people and environments that an individual perceives as threatening, leading to reduced help-seeking. For example, a previous study reported that, compared with controls, individuals with severe health anxiety were more likely to rate photos of healthy people as being less healthy, demonstrating a bias toward evaluating others as a health threat [7]. Alternatively, or in combination with avoidance behaviors, health-anxious individuals may engage in reassurance-seeking to alleviate anxiety. Both avoidance and reassurance-seeking maintain the anxiety as the individual foregoes their opportunity to become aware of their ability to cope with their perceived fear [8,9], thus preventing long-term cognitive change [10].

In addition to the physical health burden of pandemics, psychological well-being is often vulnerable. Health anxiety in particular is associated with greater levels of distress, thus warranting a greater need for psychological support [11-13]. However, during the COVID-19 pandemic, physical distancing measures used to mitigate the virus limited access to psychological care. Therefore, health care services saw service delivery transition from in-person to telehealth. Despite the shift in service delivery, additional therapy tools such as chatbots were needed to meet the increased demand for support.

Chatbots are conversational agents that hold text, speech, or visual-based conversations with users [14]. The interactions of chatbots are determined by 2 main mechanisms: artificial intelligence (AI) and decision trees. AI-based chatbots use a complex mathematical algorithm to produce specific predefined outputs based on the information input by users. Decision tree–based chatbots instead follow a prewritten script that users interact with by choosing prewritten responses. The mimicking of human conversation within the technology lends well to the use of chatbots in a mental health context. Previous studies have demonstrated that chatbot interventions improve psychological difficulties including major depressive disorder [15], panic disorder [16], posttraumatic stress disorder [17], antipsychotic medication adherence [18], and perceived stress [19].

Cognitive behavioral therapy (CBT) has the greatest evidence base for health anxiety management compared with several control conditions, including treatment as usual, medication, placebo, waitlist, support groups, non-CBT–based psychoeducation, and other psychological interventions [20,21]. CBT has been adapted for internet-based use (iCBT) in treating health anxiety and has shown similar effects to face-to-face CBT [22]. iCBT has been delivered using several digital health intervention (DHI) media such as computer programs, websites, emails, videos, mobile applications, and, more recently, chatbots. Although iCBT has been adapted to suit several digital modes of delivery to treat health anxiety, to the best of our knowledge, there has not yet been a chatbot designed to help people manage health anxiety using CBT.

CBT for health anxiety management delivered through chatbot technology may be a suitable DHI, especially in a pandemic context. A chatbot may provide an initial management intervention that reduces the burden of help-seeking individuals who may otherwise overutilize health services. Moreover, it may encourage interaction for avoidant individuals who would otherwise be reluctant to seek care, due to the presence of clinicians and anxiety-provoking settings (eg, hospitals and clinics). Although chatbots have demonstrated improvements in general well-being, few have been used to address specific clinically significant mental health disorders such as health anxiety.

## Methods

### Recruitment

**Figure 1:**
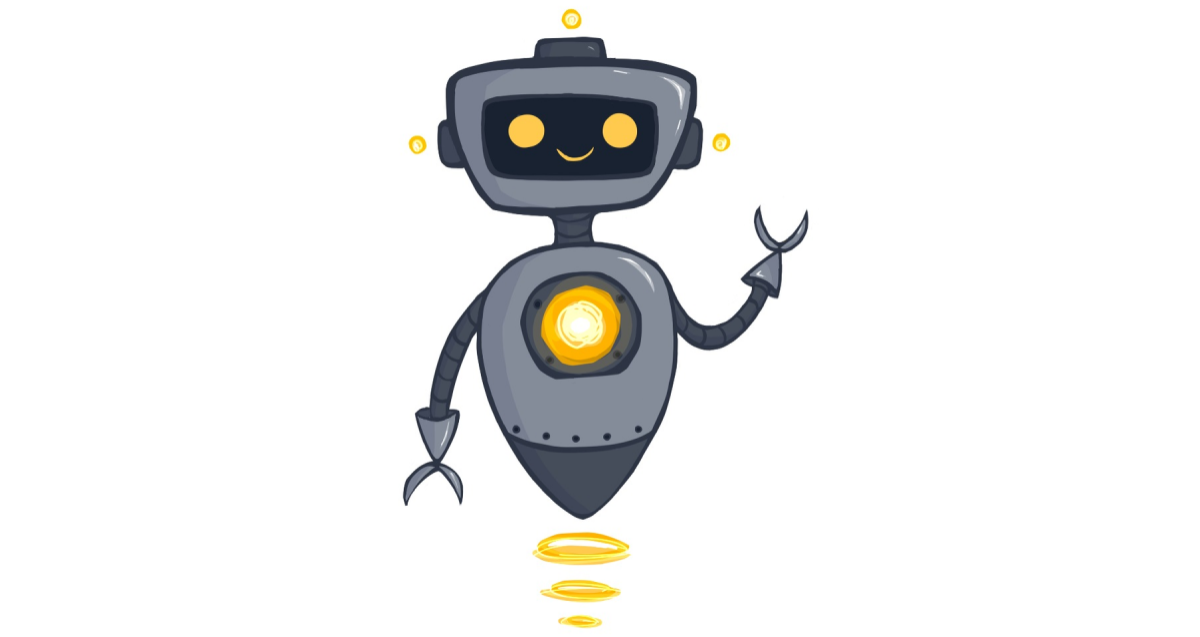
Avatar of Otis.

This mixed methods pilot study was conducted using a single group, pretest-posttest intervention framework and semistructured interviews to assess the feasibility, acceptability, engagement, and effectiveness of an automated conversational agent, “Otis” (Figure 1), as a brief health anxiety management program. Participants were recruited via Facebook and Instagram advertising, Twitter promotions by the authors’ and faculty accounts, and email invitations between May 2020 and July 2020. Participants aged 18 years and older who had access to a Facebook Messenger account and internet access that allowed for daily use of Messenger during the 14-day program were included in the study. Participants were excluded if they were not living in New Zealand or unable to understand written English to consent to study participation and complete the program.

### Ethical Considerations

The study was approved by The University of Auckland Human Participants Ethics Committee (UoA Reference: 024655). Once participants clicked on the advertising, a conversation with Otis on Facebook Messenger was opened to invite participants to read a participant information sheet. The participant information sheet outlined the inclusion criteria, study timeline, and author contact details and provided a link to an online consent form.

### Procedures

On completion of the baseline questionnaire, delivered using the web-based survey software Qualtrics [23], participants were provided a randomized user identification number to access Otis on Facebook Messenger. At the conclusion of the 14-day program or abandonment, whichever came first, participants were contacted to complete a postintervention questionnaire. Those who completed the postintervention questionnaire were invited to complete an interview with one of the researchers about their user experience and then contacted again in 12 weeks to complete a follow-up questionnaire. Participants were offered an entry in a prize draw to win one of 5 NZ $50 (US $28.36) retail vouchers per completed questionnaire. Additional entries were offered to those who completed the interview.

### Intervention

Otis was primarily a decision tree–based conversational agent or chatbot that was designed to deliver daily modules of CBT in the form of a 14-day program. Participants chatted with Otis for 5 minutes to 10 minutes per day to learn to manage health anxiety. The chatbot was developed using Chatfuel, a widely used commercial chatbot engine for Facebook Messenger, and accessed through the Facebook Messenger app or the desktop site. Previous effective CBT interventions for health anxiety have been approximately 12 modules [24,25]. Therefore, 12 modules focused on content were also chosen for Otis, with 1 module available to participants per day.

We included 2 additional modules at the start and end of the program, making the program 14 days long in total. The first module focused on introducing the intervention and collecting participant data, and the content was summarized in the final module. The content of the chatbot was derived from previous iCBT interventions and the authors’ clinical knowledge. Table 1 provides an overview of the core components that were covered in the 12 modules.

**Table 1:** Core components of the chatbot intervention.

Component	Description
Psychoeducation	Health anxious thoughts and behaviors, importance of managing health anxiety
CBT^a^ five-part model	Introduction to different components of the model and the relationships between the components
Cognitive restructuring and thinking errors	Aid identification and naming of unhelpful thinking patterns
Anxiety reduction	Mindfulness, relaxation, delaying worry exercises (eg, worry time)
Exposure	Discuss the importance of having an opportunity to endure anxiety, complete exposure ladder

^a^CBT: cognitive behavioral therapy

**Figure 2:**
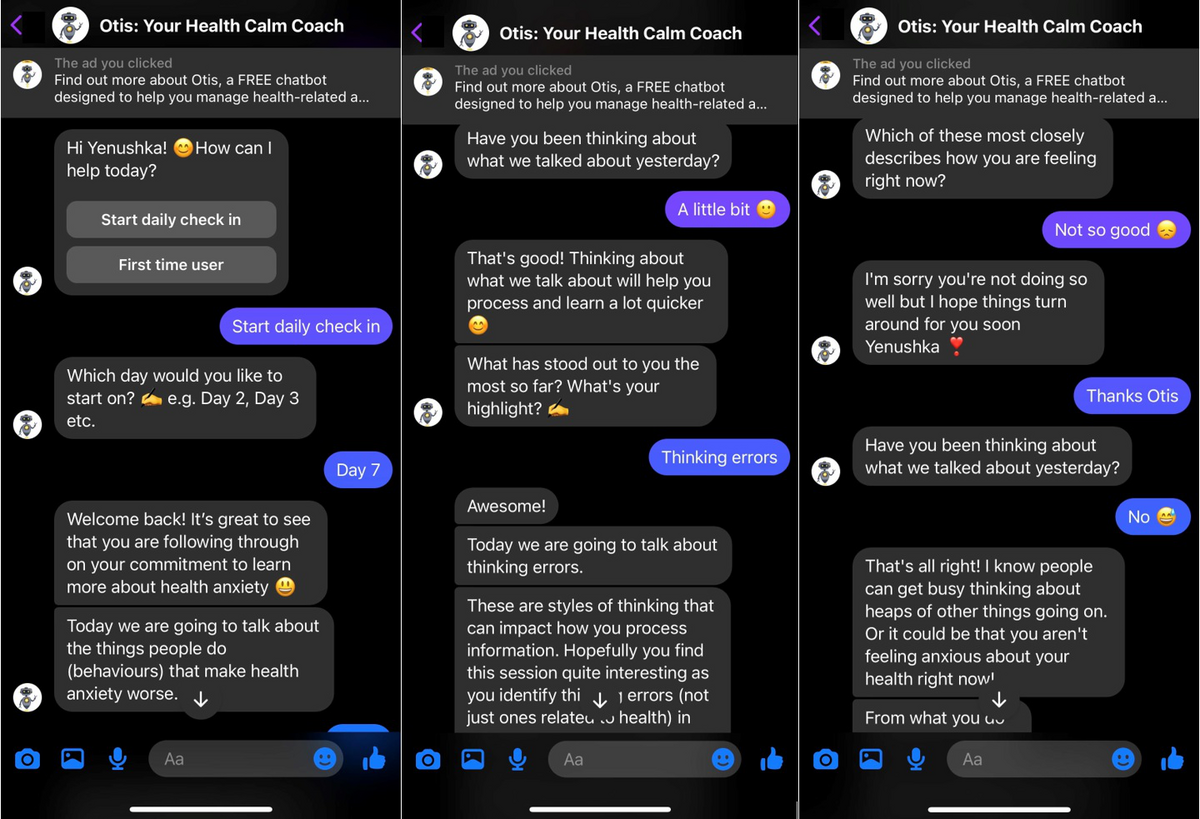
Examples of conversations with Otis.

During the initial development phase of a chatbot, it can produce crude responses that users may perceive as invalidating, robotic, or disconnected from their response [26]. Therefore, a primarily decision tree–based chatbot was chosen as the most suitable mechanism given the possibly sensitive nature of discussing mental health online. Additionally, using a decision tree–based chatbot ensured that participants received a consistent intervention. The conversations participants have within a decision tree–based chatbot are based on a prewritten script so that users follow a set conversation pathway rather than receiving a machine-learned response. Participants interacted with the chatbot by choosing predetermined text and emojis responses called “quick replies” (Figure 2). Quick replies ensured that the conversation did not deviate from the day’s content, as different replies often prompted the same response from Otis. To simulate empathy, some quick replies prompted specific validation that diverged from the pathway to form the “branches” of the tree before eventually converging back to the main script. When a message from Otis was followed by a Writing Hand emoji, this indicated to the user that they could type their own message rather than use a quick reply. Some basic AI rules were built in to reciprocate user greetings (eg, “hello,” “how are you?” “goodbye”), change notification preferences, and direct participants to crisis helplines if they asked for help or used risk words indicating suicidal ideation.

Participants were allocated 21 days to complete the 14-day program to maximize engagement and to account for fluctuating motivation and availability. On the first day, participants were able to choose a check-in time so that the chatbot triggered a notification reminder at the same time each day. If a notification had not been responded to or a participant had not completed the day’s module in full within 3 days, an additional notification was sent. A similar notification was sent if a participant had not actively used Otis for 7 days. The chatbot sent a link to the postintervention questionnaire when a participant had completed the program or 21 days following the completion of the baseline questionnaire but not the program (abandonment), whichever came first. When the program was abandoned or completed, users could no longer access the chatbot; however, the crisis line feature was available for safety. Otis was not made available to new users at the end of the study period.

### Measures

#### Demographics

At baseline, participant demographics (age, gender, ethnicity, education level, employment status, and household income), technology use, mental health service utilization, and medication use were recorded.

#### Outcome Measures

##### Short Health Anxiety Inventory

The Short Health Anxiety Inventory (SHAI-18) [27] is an 18-item self-report questionnaire that assesses the severity of health anxiety over the past 6 months. It has demonstrated comparable validity and reliability (Cronbach ɑ=.86) to its initial 64-item counterpart, the Health Anxiety Inventory [2]. Each item of the measure consists of 4 statements and requires participants to select a statement that best describes their feelings over the previous 6 months. Each statement is scored between 0 and 3 and summed, with higher scores indicating more severe health anxiety in clinical, nonclinical, and medical samples [28]. The cut-off scores of the SHAI-18 vary across studies [29-31]; however, a previous study concluded that a score of 27 and greater differentiated health anxiety from other anxiety disorders [2]. Therefore, a cut-off score of 27 was chosen for the present study.

##### Generalized Anxiety Disorder-7

The Generalized Anxiety Disorder 7-item scale (GAD-7) is a brief self-report measure that assesses the severity of anxious thoughts and behaviors over the past 2 weeks. The measure is based on the Diagnostic Manual of Mental Disorders (DSM)-IV criteria for generalized anxiety disorder, in which the severity of anxiety is calculated by summing the participant scores on each item. Scores of 5, 10, and 15 indicate mild, moderate, and severe anxiety, respectively. The GAD-7 has been validated in adult populations, with a Cronbach ɑ of .92 and high convergent and discriminant validity [32].

##### Short Intolerance of Uncertainty Scale 7

As health anxiety is correlated with intolerance of uncertainty [2,33,34], the Short Intolerance of Uncertainty Scale (IUS-12) [35], a 12-item self-report measure that assesses an individual’s response to ambiguous situations, uncertainty, and future events, was administered. For each item, participants are asked to rate the degree to which each item is characteristic of them. The ratings are weighted from 1 to 5 and summed, with higher scores indicating greater intolerance of uncertainty. The measure has demonstrated excellent internal consistency (Cronbach ɑ=.94), good test-retest reliability, and convergent and divergent validity [36].

##### Personal Well-being Domain

The Office of National Statistics Personal Well-being Domain (ONS4) is a brief, 4-item self-report assessment that measures potential changes in well-being. The ONS4 is widely used among various patient populations [36-40] and has demonstrated good internal reliability (Cronbach *α*=.90) and convergent validity [41].

##### World Health Organization-Five Well-being Index

The World Health Organization-Five Well-being Index (WHO-5) is a 5-item self-report tool that assesses an individual’s perceived positive mood, vitality, general interest, and quality of life. The raw scores range from 0 to 25 and are calculated by summing the total scores, with 0 representing the worst and 25 representing the best possible quality of life. A vast set of literature supports the validity and reliability of the measure in several populations [42-45].

#### Adherence

Facebook’s basic participant usage data were also recorded, including the number of users and rates of completion. The completion rates of the 14-day program were used as a measure of adherence (<4 days = poor adherence; <7 days = low adherence; <10 days = moderate adherence; <14 days = high adherence).

#### Reasons for Abandonment

At postintervention, participants were asked to indicate their reasons for abandoning the program if they did not complete all 14 days. From choices of lack of time, technical difficulties, boredom, no longer requiring the chatbot, and being unable to apply the skills they learned through the chatbot, participants were asked to choose the option that best described their reason for abandonment.

#### Acceptability Rating

Mixed format questions were used to assess enjoyment and satisfaction with the chatbot. The first measure used consisted of 6 statements relating to the chatbot user experience developed by the researcher (Textbox 1). Participants were required to indicate the extent to which each statement was true in their experience with using the chatbot. The scale, ranging from “not at all” to “definitely,” was weighted from 0 to 4. The ratings across items were summed, with higher scores indicating greater satisfaction and acceptability of Otis. For the second measure, participants were asked to provide an overall rating of the chatbot on a scale from 1 to 10. Open-ended questions about how Otis could be improved, what participants found most useful about Otis, and what they liked the most and least about the chatbot were included.

**Acceptability measure items used in the pilot study of Otis.**

1. It was helpful.
2. It was easy to use.
3. It was fun.
4. Otis was nice to look at.
5. It worked smoothly (eg, without major technical glitches).
6. I would like to keep it on my device.

#### Interviews

Upon completing the postintervention questionnaire, participants were invited to complete an interview with one of the researchers about their user experience. The semistructured interviews collected qualitative data about the feasibility, acceptability, and engagement of Otis. The interviewer also asked participants about the content and content delivery of the chatbot (GIFs, images, videos, text). Participants were interviewed primarily over the video calling platform Zoom [46]; however, some participants completed the interview over the phone due to connectivity difficulties in rural areas.

### Statistical Analysis

The quantitative data were analyzed using SPSS Version 26 (IBM Corp, Armonk, NY) [47]. Statistical significance was defined as *P*≤.05. Relationships between demographic characteristics and outcome measures (health anxiety, general anxiety, intolerance of uncertainty, perceived well-being, and quality of life) at a single time point were explored using *t* tests or chi-square tests. Adherence was tested by using the average completion rate and the number of participants who persisted through the program. A dose-response relationship between adherence (categorized into poor, low, moderate, and high) and change scores of the outcome measures at postintervention and 12-week follow-up were explored using 1-way analyses of variance (ANOVAs). Average participants’ satisfaction ratings were used to partially test acceptability. Finally, the effectiveness of the chatbot was tested using paired sample *t* tests of the changes in outcome measures from baseline to postintervention and follow-up. The qualitative data were manually coded by the authors.

Interviews and feedback collected during the program, at postintervention, and 12-week follow-up were included in the qualitative analysis. An inductive thematic analysis was conducted to determine factors relating to participant acceptance of and engagement with Otis. Emerging factors were then organized into themes to answer the questions of acceptability and engagement. Engagement was defined as factors related to participants’ interest and persistence with the intervention, while participants’ use and perceptions of Otis as a chatbot for health anxiety management were defined as factors related to acceptability.

## Results

### Attrition

Of the 69 participants who completed baseline measures, 35 participants (51%) completed the postintervention questionnaire, and 29 (42%) completed the 12-week follow-up. In addition, 7 participants signaled interest in and completed an interview with a researcher.

### Demographics

The final sample of 29 participants who completed all follow-up questionnaires was predominantly female (25/29, 86%), ranging in age from 21 years to 80 years old with a mean age of 37.4 (SD 15.1) years. New Zealand Europeans or Pākehā were the most common ethnic groups in the sample (20/29, 69%). Most participants (16/29, 55%) reported they participated because they wanted help to manage their anxiety, followed by a curiosity about chatbot technology (8/29, 28%). Approximately one-half (19/29, 66%) of the sample had not used any DHI for anxiety before the study. Chi-square tests revealed no significant demographic differences between those who completed baseline and those who completed all follow-up questionnaires.

### Evaluation Outcomes

Participants completed an average of 9.8 (SD 4.43) days or 70% of the chatbot intervention before the 21-day period lapsed. Of the 29 participants included in the final analysis, 12 (41%) completed the full 14-day program. The most common reason for abandonment was lack of time (14/29, 35%). See Table 2 for a summary of the reasons cited for abandoning the program. Due to a technical fault in Facebook’s data analytics system, data relating to the unique interactions participants had with Otis could not be accurately reported. The mean overall user experience was 18.24 (SD 3.38) out of a possible score of 30. The overall acceptability of the chatbot was rated favorably on a scale of 1 to 10 (mean 8.24, SD 1.80).

On average, there was a reduction in health anxiety from baseline (mean 19.93, SD 8.82) to posttreatment (mean 19.31, SD 9.02) and 12-week follow-up (mean 17.38, SD 10.03). However, this improvement did not reach significance at posttreatment (*t*_28_=0.61, *P*=.55) or follow-up (*t*_28_=1.82, *P*=.08). The same pattern was observed in the intolerance of uncertainty of participants at baseline (mean 9.69, SD 4.34), for which the reduction in these scores did not reach significance at posttreatment (mean 7.59, SD 4.38; *t*_28_=1.82, *P*=.08) or follow-up (mean 7, SD 4.99; *t*_28_=0.44, *P*=.66).

**Table 2:** Reasons for abandoning the chatbot program (n=29).

Reasons for abandonment of Otis	Results, n (%)
I didn’t have time to use the chatbot.	14 (48)
I had technical problems.	7 (24)
I didn’t feel anxious about my health, so I didn’t need it anymore.	6 (21)
I tried to put the skills into practice, but it didn’t work for me.	1 (3)
It was boring.	1 (3)

There was a significant decrease in general anxiety from baseline (mean 9.69, SD 4.38) to postintervention (mean 7.58, SD 4.38; *t*_28_=3.30, *P*=.003). The decrease in general anxiety was maintained at the 12-week follow-up (mean 7.00, SD 4.99; *t*_28_=3.26, *P*=.003). On average, self-reported personal well-being increased from baseline (mean 5.70, SD 1.30) to postintervention (mean 6.31, SD 1.06) and 12-week follow-up (mean 6.50, SD 1.55). These were significant increases at both postintervention (*t*_28_=–1.12, *P*=.03) and follow-up (*t*_28_=–1.47, *P*=.03). There was also significant improvement in self-reported quality of life at postintervention (*t*_28_=–2.39, *P*=.02) and 12-week follow-up (*t*_28_=–3.64, *P*<.001; Table 3). Paired sample *t* tests of health anxiety, intolerance of uncertainty, general anxiety, personal well-being, and quality of life for all participants (n=35) who completed the postintervention questionnaire revealed no difference in outcomes.

Paired sample *t* tests of health anxiety, intolerance of uncertainty, general anxiety, personal well-being, and quality of life for all participants (n=35) who completed the postintervention questionnaire revealed similar results (Table 4). Health anxiety did not decrease significantly from baseline (mean 19.91, SD 8.36) to postintervention (mean 19.34, SD 8.45; *t*_34_=0.65, *P*=.52) nor did intolerance of uncertainty from baseline (mean 35.63, SD 10.50) to postintervention (mean 33.40, SD 10.5; *t*_34_=1.88, *P*=.07). The decrease in general anxiety (baseline: mean 10.20, SD 4.80; postintervention: mean 8.26, SD 4.96) as measured by the GAD-7 was significant (*t*_34_=3.58, *P*<.001). As seen in the final sample, personal well-being significantly increased from baseline (mean 5.66, SD 1.26) to postintervention (mean 6.15, SD 1.05; *t*_34_=–2.11, *P*=.04), as did quality of life (baseline: mean 9.49, SD 4.35; postintervention: mean 11.09, SD 4.53; *t*_34_=–2.62, *P*=.01).

**Table 3:** Effect of the intervention on health anxiety, intolerance of uncertainty, general anxiety, personal well-being, and quality of life.

Measure	Baseline (n=29), mean (SD)	Postintervention (n=29), mean (SD)	Follow-up (n=29), mean (SD)	Baseline to postintervention		Baseline to follow-up	
				*t* (*df*)	*P* value	*t* (*df*)	*P* value
Health anxiety (SHAI-18^a^)	19.93 (8.83)	19.31 (9.03)	17.38 (10.03)	0.61 (28)	.55	–2.28 (28)	.08
Intolerance of uncertainty (IUS-12^b^)	34.10 (9.70)	31.72 (9.71)	33.38 (10.78)	1.82 (28)	.08	0.44 (28)	.66
General anxiety (GAD-7^c^)	9.69 (4.34)	7.59 (4.38)	7.00 (4.99)	3.30 (28)	.003	3.26 (28)	.003
Personal well-being (ONS4^d^)	5.70 (1.30)	6.31 (1.06)	6.50 (1.55)	–2.33 (28)	.03	–2.28 (28)	.03
Quality of life (WHO-5^e^)	9.62 (4.53)	11.31 (4.90)	12.93 (5.08)	–2.39 (28)	.02	–3.64 (28)	.001

^a^SHAI-18: Short Health Anxiety Scale.

^b^IUS-12: Short Intolerance of Uncertainty Scale.

^c^GAD-7: Generalized Anxiety Disorder 7-item Scale.

^d^ONS4: Office of National Statistics Personal Well-being Domain.

^e^WHO-5: World Health Organization Five Well-being Index.

**Table 4:** Effect of the intervention on health anxiety, intolerance of uncertainty, general anxiety, personal well-being, and quality of life for all participants who completed the postintervention questionnaire.

Measure	Baseline (n=35), mean (SD)	Postintervention, (n=35), mean (SD)	Baseline to postintervention	
			*t* (*df*)	*P* value
Health anxiety (SHAI-18^a^)	19.91 (8.36)	19.34 (8.45)	0.65 (34)	.52
Intolerance of uncertainty (IUS-12^b^)	35.63 (10.50)	33.40 (10.50)	1.88 (34)	.07
General anxiety (GAD-7^c^)	10.20 (4.80)	8.26 (4.96)	3.58 (34)	.001
Personal well-being (ONS4^d^)	5.66 (1.26)	6.15 (1.05)	–2.11 (34)	.04
Quality of life (WHO-5^e^)	9.49 (4.35)	11.09 (4.53)	–2.62 (34)	.01

^a^SHAI-18: Short Health Anxiety Scale.

^b^IUS-12: Short Intolerance of Uncertainty Scale.

^c^GAD-7: Generalized Anxiety Disorder 7-item Scale.

^d^ONS4: Office of National Statistics Personal Well-being Domain.

^e^WHO-5: World Health Organization Five Well-being Index.

One-way ANOVAs and post hoc tests (Tukey B and Games-Howell) demonstrated no significant difference in IUS-12, GAD-7, ONS4, and WHO-5 change scores among poor, low, moderate, and high adherence groups at postintervention and the 12-week follow-up. However, the SHAI-18 change scores at follow-up were significantly different between participants who moderately and poorly adhered to the program (*F*_3,25_=3.59, *P*=.02). Scatterplots were used to further investigate the correlation between change scores and the number of days completed, but no other remarkable relationships were identified.

**Figure 3:**
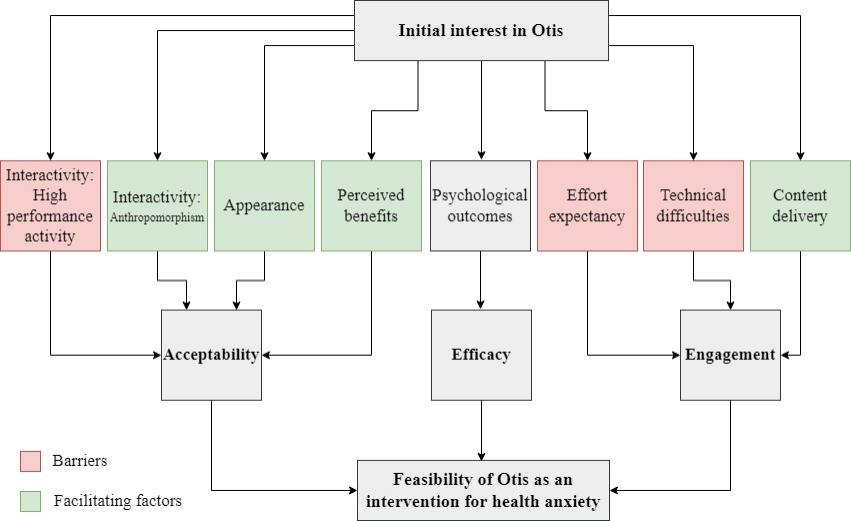
A conceptualization of the factors related to the engagement, acceptance, and feasibility of Otis.

Across the qualitative data, 3 key factors related to engagement and acceptance emerged (Figure 3). Quotes related to these factors can be found in Multimedia Appendix 1. Participants reported content delivery, technical difficulties, and effort expectancy as factors influencing their engagement with the chatbot, while themes relating to the chatbot’s appearance, perceived benefits, and interactivity (how interactive the chatbot was) influenced acceptability. Within the factors of interactivity, 2 subfactors, anthropomorphizing of Otis and high-performance expectancy, were found to distinctly affect acceptability.

#### Engagement

Regarding effort expectancy, participants reported that, although the “check-ins” were short, conversing with the chatbot daily was tedious and the expectation of participant time was too much. Social engagements, fatigue, and daily tasks were commonly cited as distractions.

Technological difficulties were cited as one of the main reasons for abandonment in the quantitative data. It was reflected in the feedback and interviews, as people discussed the frustration of Facebook and Chatfuel malfunctions and difficulties with understanding how to interact with Otis.

Regarding content delivery, the delivery of information in short “check-ins” and short messages was favored by the participants who reported that, if the information was presented in a long set of text, it would be more tiring to read and less engaging. Additionally, participants noted that the pace of information delivery was manageable and easy to comprehend.

#### Acceptance

Interactivity was assessed by the anthropomorphizing of Otis and high-performance expectancy. Regarding the anthropomorphizing of Otis, nearly all participants attributed human-like characteristics to Otis and gave Otis feedback on days 4, 11, and 14 as if it were a person. Despite the robot avatar, participants explained that the content of the conversation, use of emojis, and experience of texting made Otis feel like another “person” they were chatting with online. Regarding high-performance expectancy, although participants attributed human-like characteristics to Otis, they were also aware of the limitations of a decision tree–based chatbot. Participants described a lack of personalization that could have been achieved with “smart” or AI chatbots. Others described instances where they were unable to discuss certain topics in-depth or address them at all as they were not part of the script.

Otis’ appearance appeared to be important for the acceptance of the chatbot as an intervention for health anxiety. There appeared to be a preference for a chatbot avatar over a human or human-like design.

Regarding perceived benefits, participants reported that they found Otis helpful and made them gain greater insight into health anxiety and develop strategies to challenge their anxious thoughts. It appeared that these benefits saw participants accepting Otis as a valid intervention for health anxiety.

## Discussion

### Principal Findings

Based on a review of the literature and to the best of our knowledge, this is the first study to assess the feasibility, efficacy, acceptability, and engagement of a chatbot (Otis) for health anxiety management among adults. The results suggested that, although there was no significant reduction in health anxiety, the chatbot was accepted as an intervention for health anxiety management and was associated with decreased generalized anxiety and increased perceived anxiety management, personal well-being, and quality of life. The study identified several barriers (high performance expectancy, effort expectancy, technical difficulties) and facilitators (appearance, anthropomorphism, perceived benefits, content delivery) to the engagement and acceptability of chatbots delivering a psychological intervention.

### Comparison With Prior Work

This study found that approximately 41% of participants completed the study and that, on average, the final sample completed 70% of the intervention, which is higher than seen in most of the literature [48-50]. The high rate of adherence may indicate (1) a highly motivated sample of participants considering that most participants wanted help with managing anxiety (55%), (2) frequent phone users in the sample (>10 times per day = 35%), or (3) that the conversational nature of a chatbot is a more engaging medium than others for delivering online CBT programs.

Although Otis may be engaging, the intervention was not associated with a significant reduction in health anxiety at postintervention or the 12-week follow-up, which may indicate that CBT delivered via chatbot as a treatment for health anxiety is ineffective. This is inconsistent with what has been reported in previous studies in which CBT delivered via digital media was effective in reducing health anxiety in adult populations [22]. Instead, the results were consistent with a systematic review and meta-analysis of mental health chatbots demonstrating weak evidence for chatbots producing clinically significant psychological outcomes [26].

Failure to reach significance may also be explained by the low statistical power of the study due to a small sample size. Additionally, the results may demonstrate a floor effect during the COVID-19 pandemic, during which it was adaptive to be vigilant about health, in which case the SHAI-18 may have been measuring atypical behavior seen and encouraged during a pandemic. Therefore, although the chatbot was associated with a decrease in health anxiety, perhaps in the context of a pandemic, Otis could have no further effect in lowering health anxiety.

The qualitative results revealed that participants perceived Otis as useful in reducing their health anxiety. The inconsistency between the quantitative and qualitative results may indicate that the program was associated with improved anxiety management but that health anxiety persisted as an adaptive response to COVID-19 and New Zealand’s several lockdowns. Furthermore, only 5 participants in the study met the cut-off score (25) for health anxiety [2], which further supports the supposition of a floor effect in the remainder of the sample. Additionally, these results may reflect the perception of increased usefulness beyond the actual functionality of a chatbot that is associated with anthropomorphism [51].

To the best of our knowledge, no study has shown significantly improved anxiety compared with a control group using a CBT-based chatbot. For example, a study into Woebot’s effect on anxiety found that the information-only condition was as effective in reducing anxiety as the chatbot user group [52]. As exposure and habituation are key components in the treatment of anxiety, chatbots may not be a suitable medium, as the technology is unable to actively incorporate and encourage these components of treatment in the same way a therapist could. Otis was also associated with significant improvements in users’ personal well-being and quality of life at both postintervention and the 12-week follow-up. These results are congruent with a large body of literature demonstrating improved anxiety is linked to a better quality of life and well-being. Lockdowns have a significant impact on mental well-being and other aspects related to quality of life. Therefore, an alternative explanation for the improvement across these measures may be a result of New Zealand moving into less restrictive lockdown alert levels during the course of the study.

A systematic review of barriers to facilitators of engagement with mobile health (mHealth) interventions reported that a user’s ability to integrate the intervention into their lives impacted engagement [53]. The importance of integration is reflected in this study in which the perceived high effort expectancy of using Otis competed with other activities leading to forgetfulness or deprioritizing of use, as seen in other studies [54,55]. The themes of technical difficulties and content delivery affecting engagement with Otis reflected the results in the systematic review by Borghouts and colleagues [53]. Technical difficulties are a key issue to consider in mHealth interventions as they are often unavoidable. For example, technology is constantly updated to make improvements to the user experience. However, these updates can cause systems to crash and slow, causing disruptions to users. Disruption can significantly impact a user’s trust in the system and motivation to engage especially when in a discussion about their mental health. Finally, participants found that Otis’ short and varied content delivery (text, videos, audio, and GIFs) was engaging, which is congruent with other studies assessing the effects of content delivery on overall user experience [56]. Despite relatively high engagement and reported acceptability, participants did not consider Otis a substitute for a health professional, in line with previous research that showed a preference for CBT and medication in the treatment of health anxiety over iCBT [57].

An unexpected finding in this study was participants’ preference for a nonhuman chatbot avatar. Though previous work suggests that anthropomorphism is amplified by human-looking avatars [58], even in the absence of a human avatar, users responded socially to Otis. A recent study reported that users’ perceived trustworthiness and affinity were lower when a realistic digital avatar was used as compared with a human being but remained unaffected by the knowledge of whether a chatbot was controlled by a person or AI [59]. Although the aforementioned study did not compare human and nonhuman avatars, its findings indicate that appearance makes a significant difference to the user experience. Participants in this study reported feelings of mistrust and worries of judgment as they would have with a real human if Otis were not a robot figure, thus being a barrier to acceptance as an intervention for health anxiety. These findings are in direct conflict with previous research suggesting that, if users were to apply the same social rules to their interactions with a chatbot, it may improve a user’s perception of social presence [60].

A study that appears to be in partial support of the findings of this study reported that too many anthropomorphic characteristics in a chatbot avatar set up high expectations, leading to user disappointment [61], thus supporting the idea that high expectations of functionality can lead to feelings of disappointment and frustration for participants. Upgrading Otis and other chatbots to AI-based chatbots that can understand and better process user input may meet the expectations of users in the future. The results presented in this study and the existing literature should be considered in work with “digital humans,” which aim to make digital interactions more personalized and lifelike. Consideration should be given for which contexts and populations the highly anthropomorphic digital human characters would be suitable.

### Limitations

The observational design of this study limited the ability to imply causation. Additionally, the results may have been confounded by unidentifiable variables such as psychological input outside of the intervention and changes in COVID-19 restrictions. Although the plethora of pilot and feasibility studies within the field of mHealth is a common criticism, the use of randomized controlled trials only provides evidence of the efficacy of a digital intervention at a certain point in time. Pilot and feasibility studies instead grow understanding of factors involved in digital interactions to be implemented in evolving technology. The sample was mostly New Zealand European and highly educated, thus limiting the generalizability of the findings across the population. Additionally, bias within the interpretation of the qualitative results, a common critique of qualitative research [62], is another limitation of the study.

### Conclusions

In conclusion, Otis, a CBT-based chatbot, may be a feasible intervention for the management but not treatment of health anxiety among adults. The study found that, although there was no significant improvement in health anxiety, participants reported benefiting from the intervention, which is further evidenced by improvements in general anxiety, personal well-being, and quality of life. The results suggest that mental health chatbots are a feasible supplementary treatment that can alleviate strain on psychological services. Therefore, future studies should continue to evaluate the feasibility of chatbots in this space as the technology develops. This pilot study makes a unique contribution to the understanding of digital interactions, health anxiety, and the potential of chatbots in mental health care. Future studies in the area should continue to investigate the “therapeutic relationship” between people and technology to aid the development of mHealth, thereby facilitating the democratization of mental health services.

## Appendix

Multimedia Appendix 1 Participant quotes regarding engagement and acceptance of Otis as an intervention for health anxiety management.

## Abbreviations

AI: artificial intelligence
ANOVA: analysis of variance
CBT: cognitive behavioral therapy
DHI: digital health intervention
DSM: Diagnostic Manual of Mental Disorders
GAD-7: Generalized Anxiety Disorder 7-item Scale
iCBT: internet-based cognitive behavioral therapy
IUS-12: Intolerance of Uncertainty Scale
mHealth: mobile health
ONS4: Office of National Statistics Personal Well-being Domain
SHAI-18: Short Health Anxiety Scale
WHO-5: World Health Organization Five Well-being Index
