# Supplementary material for: A Cognitive Behavioral Therapy Chatbot (Otis) for Health Anxiety Management: Mixed Methods Pilot Study
Source: JMIR Form Res. 2022 Oct 20;6(10):e37877. doi: 10.2196/37877 (PMC9586257; doi:10.2196/37877)
Supplement: Multimedia Appendix 1 [file formative_v6i10e37877_app1.docx]

Appendix 1. Participant quotes regarding engagement and acceptance of Otis as an intervention for health anxiety management

**Engagement**

| **Factor** | **Participant quotes** |
| --- | --- |
| Effort expectancy | *“It’s good! I think just hard to commit to every day which is why I have missed some days - hard when schedules change to keep it up i.e. weekend” -* Participant 12, day 14 feedback    *“It should be available to use whenever you want. Some days I feel like chatting longer and some days I just don’t have time to” -* Participant 13, post-intervention feedback    *“[What Otis should improve] if Otis was able to check in with me about every other day perhaps for a slightly longer session that would suit my attention span and dedication to it”* - Participant 2, post-intervention feedback |
| Technical difficulties | *“It felt like a real person even though I know it's a chatbot. Except.... when it started glitching in the last 3 or so days. Then I was irritated when it didn't work properly”* – Participant 23, post-intervention feedback  *“I kept getting double notifications from the messenger but also fb! But I think this was a glitch of my phone as opposed to Otis”* – Participant 11, day 4 feedback  *“[What you liked least about Otis] That my phone kept going to sleep when trying the relaxation exercises if I wasn't looking at the screen* – Participant 10, post-intervention feedback  *“There should be a way/command to 'reset' the day if Otis gets stalled for some reason. We shouldn’t have to 'talk to a human' to fix this”* – Participant 24 |
| Content delivery | *“[What I liked about Otis was] conversational and interactive presenting of information, humour and it usually only took about 5 to 10 minutes of my time on any given day”* - Participant 18, post-intervention feedback    *“I’ve really appreciated and enjoyed how everything was broken down into simple and understandable comments/info. I love that it’s interactive”* - Participant 34, day 11 feedback    *“[What I liked about Otis was] the way he got us to interact rather than us just taking in information”* - Participant 8, post-intervention feedback  *“The information was presented in a good progression/sequence”* - Participant 17, day 14 feedback |

**Acceptance**

| **Factors** | **Participant quotes** |
| --- | --- |
| Interactivity: Anthromorphisation of Otis | *“If it was sending multiple chats, it would take a pause in between, I guess so you could read it but I think that definitely made it feel more conversational because it kind of felt like somebody was thinking about it on the other end” -* Participant 2, interview    *“I liked imagining he was ‘real’, and that he was actually keeping me company and trying to help me” -* Participant 15, post-intervention feedback    *“Otis is really cute and I enjoy the response options. The conversations flow smoothly and really mimic that of texting!”* - Participant 43, day 11 feedback |
| Interactivity: High performance expectancy | *“Offering options that actually reflect the span of responses would be more engaging” -* Participant 27, day 11 feedback    *“He [Otis] just needs to get smarter so he can react when people don’t quite follow the script or get stuck”* - Participant 45, post-intervention feedback.    *“I felt like if I wanted to I could not explore my health anxiety as much than with an actual person as I know the chatbot would have had a script response to follow”* – Participant 12, post-intervention feedback |
| Appearance | *“If Otis was some kind of like humanoid character or even a photo of someone, I think you’d kind of launch a lot of your own prejudice onto Otis because it could look like someone you know, even like an ex of yours of something, I don’t think you’d be that inclined to talk to Otis… I think it’s nice to have like quite a cartoonish, friendly representation, whether that’s an animal or a robot and for me personally if it's not a human”* - Participant 7, interview    *“I would have been probably more, um, sceptical if it was a person. Yeah, I think if he was a person, I would have had to establish more trust with them before I was willing to discuss my health with them online…. I think I would have probably considered it more, signing up I guess, because I would have been like ‘is… does this person or the avatar of the person look like somebody I want to engage with?’… ‘Do they look trustworthy’ kind of thing” -* Participant 4, interview.    *“I think if he was a human, it wouldn’t be as much fun. Right? So, the fact that he’s a chatbot feels… it gives like a little bit of safety because it’s like we’re talking about serious things but there’s this cute little robot jumping up and down telling me things. I think that adds to the experience and I think it also serves to remind you that it is a chatbot” -* Participant 1, interview |
| Perceived benefits | *“I feel like I discussed a lot and was educated into knowing more about this topic without having my health anxiety or just anxiety triggered. I found everything really approachable and helpful” -* Participant 34, day 14 feedback  *“[What I enjoyed most about Otis] for me it was that I’m not the only one that feels the way I do. Understanding that the way I think and operate is not normal and that there is a name for it created an epiphany for me that gave me the confidence to believe that the techniques you were offering would work because I wasn’t the only one”* - Participant 10, post-intervention feedback  *“[What I learned from Otis] how we are having physical sensations/symptoms all the time, and they usually just go away and overly focussing on them and stressing out about those feelings increases heart rate and can intensify symptoms”* - Participant 21, post-intervention feedback |
